# The Identification of Admixture Patterns Could Refine Pharmacogenetic Counseling: Analysis of a Population-Based Sample in Mexico

**DOI:** 10.3389/fphar.2020.00324

**Published:** 2020-04-22

**Authors:** José Jaime Martínez-Magaña, Alma Delia Genis-Mendoza, Jorge Ameth Villatoro Velázquez, Beatriz Camarena, Raul Martín del Campo Sanchez, Clara Fleiz Bautista, Marycarmen Bustos Gamiño, Esbehidy Reséndiz, Alejandro Aguilar, María Elena Medina-Mora, Humberto Nicolini

**Affiliations:** ^1^ Laboratorio de Genómica de Enfermedades Psiquiátricas y Neurodegenerativas, Instituto Nacional de Medicina Genómica (INMEGEN), Mexico City, Mexico; ^2^ Hospital Psiquiátrico Infantil “Juan N. Navarro,” Servicios de Atención Psiquiátrica, Mexico City, Mexico; ^3^ Unidad de Encuestas y Análisis de Datos, Insituto Nacional de Psiquiatría Ramón de la Fuente Muñiz (INPRFM); ^4^ Global Studies Seminar, Faculty of Medicine, National Autonomous University of Mexico (UNAM), Mexico City, Mexico; ^5^ Laboratorio de Farmacogenética, Insituto Nacional de Psiquiatría Ramón de la Fuente Muñiz (INPRFM), Mexico City, Mexico

**Keywords:** Mexican population-based sample, MxGDAR/Encodat, pharmacogenetics, genomics, admixture

## Abstract

Pharmacogenetic analysis has generated translational data that could be applied to guide treatments according to individual genetic variations. However, pharmacogenetic counseling in some *mestizo* (admixed) populations may require tailoring to different patterns of admixture. The identification and clustering of individuals with related admixture patterns in such populations could help to refine the practice of pharmacogenetic counseling. This study identifies related groups in a highly admixed population-based sample from Mexico, and analyzes the differential distribution of actionable pharmacogenetic variants. A subsample of 1728 individuals from the Mexican Genomic Database for Addiction Research (MxGDAR/Encodat) was analyzed. Genotyping was performed with the commercial PsychArray BeadChip, genome-wide ancestry was estimated using EIGENSOFT, and model-based clustering was applied to defined admixture groups. Actionable pharmacogenetic variants were identified with a query to the Pharmacogenomics Knowledge Base (PharmGKB) database, and functional prediction using the Variant Effect Predictor (VEP). Allele frequencies were compared with chi-square tests and differentiation was estimated by F_ST_. Seven admixture groups were identified in Mexico. Some, like Group 1, Group 4, and Group 5, were found exclusively in certain geographic areas. More than 90% of the individuals, in some groups (Group 1, Group 4 and Group 5) were found in the Central-East and Southeast region of the country. *MTRR* p.I49M, *ABCG2* p.Q141K*, CHRNA5* p.D398N, *SLCO2B1* rs2851069 show a low degree of differentiation between admixture groups. *ANKK1* p.G318R and p.H90R, had the lowest allele frequency of Group 1. The reduction in these alleles reduces the risk of toxicity from anticancer and antihypercholesterolemic drugs. Our analysis identified different admixture patterns and described how they could be used to refine the practice of pharmacogenetic counseling for this admixed population.

## Introduction

In recent years, pharmacogenetic (PGx) studies have generated substantial information that is useful in clinical settings (Relling and Evans, 2015; Van Der Wouden et al., 2016; Verbelen et al., 2017). PGx variation influences the efficacy and toxicity of drugs through the alteration of pharmacodynamic or pharmacokinetic processes. Pharmacogenetic studies have uncovered many relationships between drugs and specific genes, but not all of this information can be used to implement PGx-based treatment guidelines. Various initiatives have attempted to compile information and develop PGx evidence-based drug dosing guidelines, like the Pharmacogenomics Knowledge Base (PharmGKB; (Thorn et al., 2013)). The PharmGKB classifies pharmacogenes into four different evidence levels (Barbarino et al., 2018), where pharmacogenes at higher levels are termed “actionable”–that is, they can be used in treatment guidelines for PGx-based counseling.

Most pharmacogenetic studies have been carried out on individuals with a low degree of admixture. Actionable allele frequencies are dependent on ancestry, and such differences must be taken into account in clinical counseling (Abdul Jalil et al., 2015; Mizzi et al., 2016; Goh et al., 2017). A recent analysis of PGx variation across 26 global populations using data from Phase 3 of the 1000 Genomes Project identified clusters of individuals by continent, with high degrees of differentiation even among continental populations (Wright et al., 2018). Researchers have expressed concerns about the assignment of ancestry in PGx analysis, principally because of its potential impact on the differentiation of genetic variants among continental populations (Zhang and Finkelstein, 2019).

Mexico has a differential pattern of ancestry, made up primarily of three populations: Native American, European, and African (Wang et al., 2008; Moreno-Estrada et al., 2014). Its genomic ancestry has been divided into Native American (NA) and Mexican Mestizo (MM) classifications (Silva-Zolezzi et al., 2009). Differences have been reported between these two groups in actionable PGx variants, including greater allele frequency of *VKORC1* (rs8050894), *CYP2B6* (rs2279343), and *CYP3A5* (rs776746) in the NA population, and of *CYP2C19* (rs4244285), *CYP2C9* (rs1799853, rs1057910), *NAT2* (rs179930), *SLCO1B1* (rs4149015), and *APOE* (rs7412) in the MM population (Gonzalez-Covarrubias et al., 2019). The MM population is the largest in Mexico, and the prevalence of admixture patterns in this population is high. Grouping all MM individuals together could thus hide differences in allele frequencies. These differences have not been estimated for the MM population based on the degree of admixture or the distribution of the genome-wide global ancestry. However, an analysis of the admixture patterns and clustering of individuals with similarities could improve pharmacogenetic counseling for individuals in this population. The aim of the present study is thus to analyze global admixture patterns in a population-based Mexican sample in order to assess their impact on actionable pharmacogenetic variants.

## Methods

### Participants

**Figure 1:**
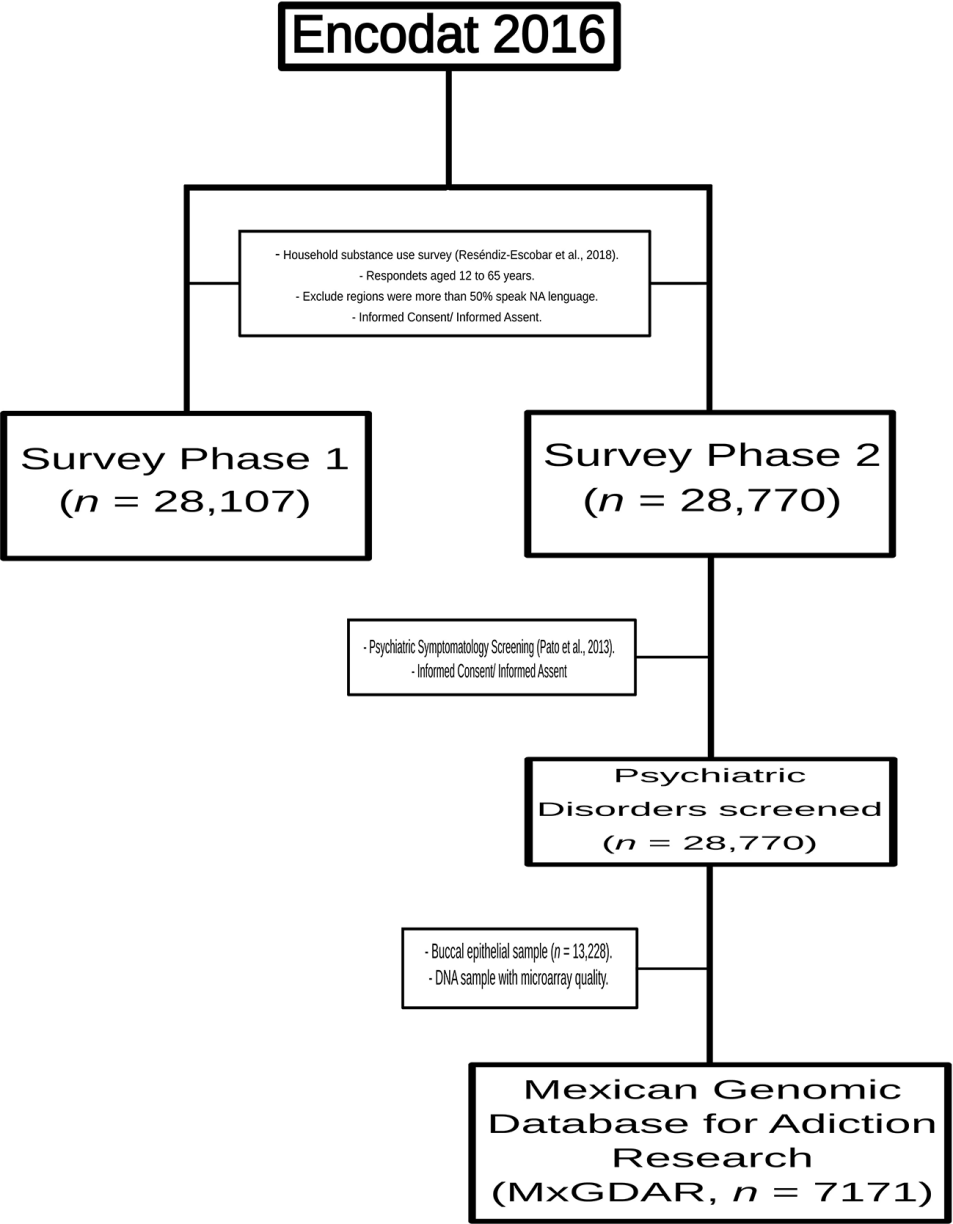
Sampling scheme for the Mexican Genomic Database for Addiction Research (MxGDAR/Encodat).

This study analyzed a subsample of the Mexican Genomic Database for Addiction Research (MxGDAR/Encodat), derived from the Mexican National Survey of Tobacco, Alcohol, and Drug use (Villatoro-Velázquez et al., 2017). All of the geographic regions where more than half of the population speaks a Native American (NA) language were excluded (Villarreal, 2014). The survey was carried out in two phases, with the sampling performed in the second phase. There were questionnaires in each phase: the first focused on sociodemographic, social, and interpersonal information, with a section on patterns of alcohol, tobacco, and drug use (Reséndiz-Escobar et al., 2018), and the second on screening for psychiatric symptomatology (Pato et al., 2013). In the second phase, a sample of buccal epithelial cells was also collected (**Figure 1**). All of the protocols in this study were approved by the Research Ethics Committees of the Instituto Nacional de Psiquiatría Ramón de la Fuente Muñiz (Approval No. CEI/C/083/2015) and the Instituto Nacional de Medicina Genómica (Approval No. 01/2017/I). The aims of the study were explained to each participant, and each was informed that they could end their participation at any time. All participants provided written informed consent; assent for a minor participant was obtained both from the participant and from a parent or legal guardian.

### DNA Extraction, Microarray Genotyping, and Quality Control

DNA was extracted using a commercial modified salting-out procedure (Qiagen, USA), according to the manufacturer’s instructions. DNA extraction quality and integrity were evaluated by analysis with a NanoDrop spectrophotometer (Thermofisher, USA) and 2% agarose gel. The MxGDAR/Encodat database included 7171 of the 13,228 buccal epithelial samples collected (54.21%) that met the following quality criteria: i) 230/260 and 260/230 ratios > 1.8, ii) concentration > 50 ng/µL, and iii) no signs of DNA degradation. The DNA extraction was divided evenly between the Laboratorio de Genómica de Enfermedades Psiquiátricas y Neurodegenerativas of the Instituto Nacional de Medicina Genómica and the Departamento de Farmacogenómica of the Instituto Nacional de Psiquiatría Ramón de la Fuente Muñiz. We found no difference in the quality of microarray samples between the laboratories; the insufficient quality of half of the samples could have been an effect of problems in sample collection procedures. In a household survey like the Encodat 2016, collecting biological samples is problematic because it cannot be done under the controlled conditions of a clinical environment (Gudiseva et al., 2016).

Genotyping was performed with the commercial microarray PsychArray BeadChip (Illumina, USA), according to the manufacturer’s instructions. In this preliminary analysis we genotyped a total of 1728 samples, with a random sampling that included at least 15 individuals from each of the 32 states in Mexico. The fluorescent intensities were read with the iScan system (Illumina, USA) and converted to genotype calls with Genome Studio software (Illumina, USA). Genotyping was carried out at the Unidad de Alta Tecnología para Expresión y Microarreglos of the Instituto Nacional de Medicina Genómica. The quality control of the genotyped data was performed using Plink (Purcell et al., 2007). Single nucleotide polymorphisms (SNPs) were excluded if they did not meet the following criteria: i) no-call rate > 5%, ii) minor allele frequency (MAF) < 5%, iii) *p*-value < 1E-06 in a chi-square test for Hardy-Weinberg equilibrium, or iv) they were duplicates. Individuals were excluded if they had no call rate > 5%. To identify cryptic familial relationships, we performed an identity-by-state (IBS): all individuals with an IBS > 1.6 were marked and those with the lower genotyping rate were excluded. This quality control left 1657 individuals for analysis.

### Global Ancestry Estimations and Model-Based Admixture Clustering

Global ancestry estimations were performed with a genome-wide approach. For this purpose, the SNPs of the 1657 individuals that remained after quality filters were then filtered for independence. The linkage disequilibrium (LD) pruning algorithm was implemented in Plink, with a window size of 50 kb, a step size of 5, and a variance inflation factor (VIF) of 2. LD pruning left a total of 104,726 SNPs for analysis. The database was then merged with the SNPs of the Human Genome Diversity Project (HGDP; (Cavalli-Sforza, 2005)), and the SNPs not present in either database were excluded, leaving a total of 25,562 SNPs for genome-wide global ancestry estimations. EIGENSOFT (Price et al., 2006), was used to calculate ten global ancestry components. In this estimation, 133 individuals with values greater than three standard deviations were excluded, leaving 1524 individuals for analysis. The 133 excluded individuals had admixture proportions of European ancestry greater than 96%, as estimated through a model-based analysis with ADMIXTURE software (Scheet and Stephens, 2006). We removed these individuals, because we also filtered the communities with higher NA ancestry during the sampling procedure, to not deviate the calculation of admixture patterns to the founders’ populations. The sociodemographic characteristics of the sample are shown in **Table 1**.

**Table 1:** Sociodemographic characteristics of the subsample.

	Subsample MxGDAR/Encodat (*n* = 1525)
**Age (years) (*SD*)**	38.34 (14.46)
**Gender**	
Male	652 (42.75%)
Female	873 (57.25%)
**Marital Status**	
Married	645 (42.30%)
Cohabiting	289 (18.95%)
Separated	82 (5.38%)
Divorced	30 (1.98%)
Widowed	56 (3.67%)
Single	423 (27.74%)
**Occupation**	
Professional	30 (1.96%)
Teacher	13 (0.85%)
Company Director	3 (0.19%)
Small Business Owner	85 (5.57%)
Office Worker	78 (5.11%)
Skilled worker	73 (4.79%)
Unskilled worker	227 (18.16%)
Farm Worker	24 (1.57%)
Farmer	71 (4.66%)
Unemployed	68 (4.46%)
Student	115 (7.54%)
Housewife	519 (34.03%)
Retired	30 (1.98%)
Other	139 (9.04%)

Next, a model-based clustering algorithm using mclust software (Scrucca et al., 2016) carried out Gaussian mixture modeling to identify admixture groups in the subsample of 1524 individuals. A geographic distribution of the groups was then determined for the following regions of Mexico: i) Northwest (Baja California, Baja California Sur, Nayarit, Sinaloa, and Sonora), ii) North (Coahuila, Chihuahua, Durango, Nuevo León, San Luis Potosí, Tamaulipas, and Zacatecas), iii) Central-West (Aguascalientes, Colima, Jalisco, and Michoacán), iv) Central-East (Mexico City, Guerrero, Hidalgo, Estado de México, Morelos, Puebla, Quéretaro, and Tlaxcala), and v) Southeast (Campeche, Chiapas, Oaxaca, Quintana Roo, Tabasco, Veracruz, and Yucatán) (Borges et al., 2018).

### Annotation and Actionable Pharmacogenetic Variant Analysis in Admixture Subgroups

Variants in genes with actionable pharmacogenetic effects were then identified using the hg19 coordinates for those genes. Genes were included for evidence levels of 1 to 2, according to the classification of the PharmGKB (Barbarino et al., 2018), a searchable pharmacogenetic knowledge database that categorizes genes according to levels of evidence for alterations in their response to different drugs, and that provides pharmacogenetic treatment guidance where those levels are high. For genes with evidence levels of 1 or 2, PsychArray has a total of 7955 single-nucleotide variants (65.83% of the total reported in the 1000 Genomes Database), of which 5809 in our population (73.02%) had an MAF < 0.05, 185 (23.33%) were excluded for a low call rate, and 11 (0.14%) were excluded for Hardy-Weinberger disequilibrium (**Supplementary Table 1** contains the list of variants lost in these filtering steps). Some important pharmacogenetic variation was lost during these filtering processes, like those found in *CYP2D6* (evidence level 1A). Of the twelve variants design on *CYP2D6* that could be found in the PsychArray, 10 were lost in the MAF, and 2 in the call rate filtering.

We extracted the variants in the genes with actionable pharmacogenetic effect with annotations in the Ensembl Variant Effect Predictor (McLaren et al., 2016), and extracted all the variants that were missense (classifying these as damaging if a damaging effect was predicted by both SIFT and Polyphen2, but benign if only one algorithm predicted a damaging effect), synonymous, or annotated with a regulatory region. For regulatory variants, we extracted those with an annotated regulatory region based in the ENCODE identifier. A manual search was performed in PharmGKB for the evidence level for the benign missense, synonymous, and regulatory variants; for annotated variants a search was performed in PubMed for reports of at least 10 associations with drug-variant relationships. The global allele frequency of each actionable pharmacogenetic missense variant in the MxGDAR/Encodat was compared with those identified in the Genome Aggregation Database (gnomAD; (Kobayashi et al., 2017)) using a chi-square test and a delta de MAF calculation (dMAF). The degree of differentiation of variants between the admixture subgroups was analyzed with Wright’s fixation index (F_ST_; (Patterson et al., 2006)).

## Results

### Analysis of the Regional Distribution of Admixture Subgroups

**Figure 2:**
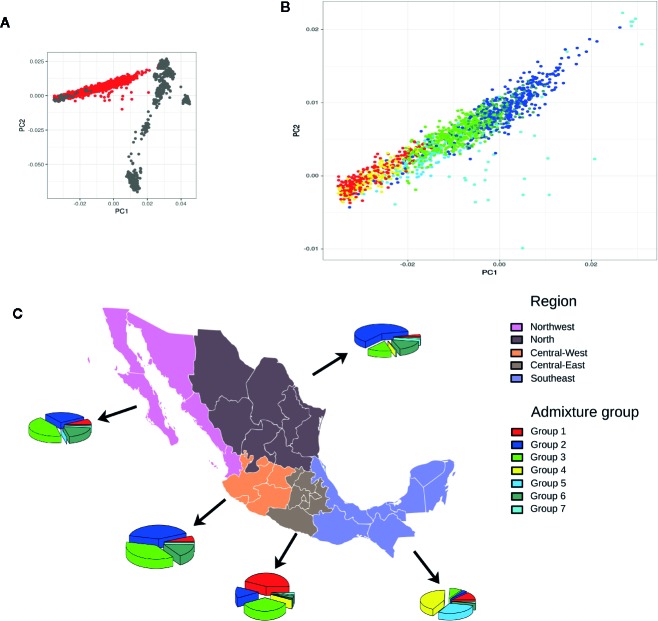
Admixture analysis of the MxGDAR/Encodat. Panels: **(A)** Principal component analysis with the Human Genome Diversity Project Database (HGDP) admixture reference; individuals from the MxGDAR/Encodat in red and individuals from the HGDP database in gray; **(B)** Plotting of the individuals from the MxGDAR/Encodat (those in red in panel **A**), color according to the admixture group identified by Gaussian model-based clustering; **(C)** Analysis of the geographical distribution of the identified admixture subgroups in each region. Each pie represents the percentages of admixture groups identified in that particular region.

An analysis of global ancestry found the distribution of European to Native American ancestry of individuals in the MxGDAR/Encodat (**Figure 2A**), and an analysis of admixture groups detected seven groups (**Figure 2B**). Groups 2 and 3 show the greatest frequency, with a total of 821 (53.87%) individuals in the two groups (**Table 2**). Groups 2, 3, and 7 have a heterogeneous distribution in different regions, with no local concentration. Groups 4 and 5, with a total of 270 (17.72%) individuals, are concentrated in the Southeast, with more than 90% of the individuals in each group in that region (**Figure 2C**). The 170 individuals in Group 1 (72.96%) are in the Central-East and Southeast, and the 160 (94.67%) in Group 6 are in the Northwest, North, and Central-West regions. Groups 1 and 4 are the most closely related to the Native American population (**Figure 2B**).

**Table 2:** Distribution of admixture groups in Mexico by region.

Region^1^	Group 1	Group 2	Group 3	Group 4	Group 5	Group 6	Group 7	Total
**Northwest**	7 (3.68)	116 (30.77)	27 (6.08)	1 (0.64)	1 (0.88)	33 (19.53)	5 (16.12)	190 (12.47)
**North**	39 (8.82)	114 (30.24)	192 (43.24)	0 (0.00)	2 (1.77)	89 (52.66)	6 (19.35)	442 (29.00)
**Central-West**	17 (7.30)	97 (25.73)	94 (21.17)	0 (0.00)	0 (0.00)	38 (22.49)	1 (3.23)	247 (16.21)
**Central-East**	123 (52.79)	38 (10.08)	101 (22.75)	1 (0.64)	3 (2.65)	8 (4.73)	8 (25.81)	282 (18.50)
**Southeast**	47 (20.17)	12 (3.12)	30 (6.76)	155 (98.73)	107 (94.69)	1 (0.59)	11 (35.48)	363 (23.82)
Total	233 (15.29)	377 (24.74)	444 (29.14)	157 (10.30)	113 (7.41)	169 (11.09)	31 (2.03)	1524 (100.00)

^1^States included in each region: Northwest: Baja California, Baja California Sur, Nayarit, Sinaloa, and Sonora; North: Coahuila, Chihuahua, Durango, Nuevo León, San Luis Potosí, Tamaulipas, and Zacatecas; Central-West: Aguascalientes, Colima, Jalisco, and Michoacán; Central-East: Mexico City, Guerrero, Hidalgo, Estado de México, Morelos, Puebla, Quéretaro, and Tlaxcala; and Southeast: Campeche, Chiapas, Oaxaca, Quintana Roo, Tabasco, Veracruz, and Yucatán.

### Analysis of Actionable Pharmacogenetic Missense Damaging Variants

Analysis of the actionable pharmacogenetic missense variants found a total of 32 variants in 31 genes, four of which have a PharmGkB evidence level of 1 (**Table 3**): SLCO1B1*5, NUDT15*3, and CYP4F2*3 with evidence level 1A, and *XPC* p.Q939K with level 1B. The difference in minor allele frequency (dMAF) in these variants between the individuals in the MxGDAR/Encodat and the gnomAD range from -0.27 to -0.36 and are statistically significant. The variant with the greatest difference, -0.36, was *XPC* p.Q939K. NUDT15*3 was the only variant with a greater allele frequency in the MxGDAR/Encodat than in the gnomAD, with a difference of 0.45.

**Table 3:** Missense damaging variants in pharmacogenes with pharmGkB evidence Level 1.

Evidence 1A											
Chr	bp	Gene	SNP	Alleles	Synonymous	Amino acid change	MAF^1^	gMAF^2^	ΔMAF^3^	Drug	Effect on Drug
12	21331549	*SLCO1B1*	rs4149056	T > C	SLCO1B1*5	V174A	0.1060	0.1326	-.0266	Simvastatin	Higher risk of myopathy
13	48619855	*NUDT15*	rs116855232	C > T	NUDT15*3	R139C	0.0733	0.0281	0.0452	Purine-based compounds	Higher risk of leukopenia, neutropenia, or alopecia
19	15990431	*CYP4F2*	rs2108622	C > T	CYP4F2*3	V433M	0.1920	0.2735	-.0815	Warfarin	Higher dose
**Evidence 1B**											
**Chr**	**bp**	**Gene**	**SNP**	**Alleles**	**Synonymous**	**Amino acid change**	**MAF**	**gMAF**	**ΔMAF**	**Drug**	**Effect on Drug**
3	14187449	*XPC*	rs2228001	G > T	NR	Q939K	0.2601	0.6359	-.3758	Platinum-based compounds	Decreased risk for toxicity

^1^MAF, Minor allele frequency in the MxGDAR/Encodat. ^2^gMAF, Global minor allele frequency (MAF) reported by the gnomAD. ^3^ΔMAF, difference between MxDAR/Encodat individuals and gMAF.

There were 28 variants at the PharmGkB evidence level 2 (**Table 4**), 13 of which were at level 2A (*MTHFR* p.A22V, *FCGR2A* p.H166R, UGT1A1*6, *ADRB2* p.G71R, ABCB1*2, NAT2*6, CYP2C8*3, *KCNJ11* p.K23E, *GSTP1* p.I188V, *VDR* p.M51T, *NQO1* p.P187S, *APOE-E2*, and *COMT* p.V158M), and 15 at level 2B (*EPHX1* p.Y113H and p.H139R, *UMPS* p.G213A, *ADD1* p.G460W, UGT2B15*2, *MTRR* p.I49M, *OPRM1* p.N40D, *SOD2* p.V16A, *CHRNA5* p.D398N, *GP1BA* p.T161M, *XRCC1* p.Q399R, *ERCC1* p.Q506K, *ITPA* p.P32T, *CBR3* p.V244M, and *PNPLA3* p.I148M). There were differences in allele frequencies ranging from -0.45 to 0.18 between the MxGDAR/Encodat and the gnomAD. The *NQO1* p.P187S variant showed the greatest positive difference in minor allele frequency (dMAF = 0.1835) in the MxGDAR/Encodat, and the *XRCC1* p.Q399R the greatest negative difference (dMAF = -0.45). The variants *MTHFR* p.A22V, *GSTP1* p.I188V, *NQO1* p.P187S, *UMPS* p.G213A, *ERCC1* p.Q506K, and *PNPLA3* p.I148M showed the largest positive differences in the MXGDAR/Encodat, and NAT2*6, *KCNJ11* p.K23E, *VDR* p.M51T, *EPHX1* p.Y113H, UGT2B15*2, *MTRR* p.I49M, *SOD2* p.V16A, *CHRNA5* p.D398N, *XRCC1* p.Q399R, and *CBR3* p.V244M the largest negative differences.

**Table 4:** Missense damaging variants in pharmacogenes with pharmGkB evidence level of 2.

Evidence 2A											
Chr	Position	Gene	SNP	Alleles	Synonymous	Amino acid change	MAF^1^	gMAF^2^	ΔMAF^3^	Drug	Effect on Drug
1	11856378	*MTHFR*	rs1801133	G > A	NR	A222V	0.4854	0.3085	0.1769	Methotrexate and platinum-based compunds	Higher risk of toxicity and decreased response
	**161479745**	***FCGR2A***	**rs1801274**	**A > G**	**NA**	**H166R**	**0.4810**	**0.4791**	**0.0019**	**Trastuzumab**	**Decreased response^4^**
2	234669144	*UGT1A1*	rs4148323	G > A	UGT1A1*6	G71R	0.0312	0.0221	0.0091	Irinotecan	Increased risk of neutropenia
5	148206440	*ADRB2*	rs1042713	G > A	NR	G16R	0.4453	0.4208	0.0245	Salbutamol/Salmeterol	Decreased response
7	87160561	*ABCB1*	rs2032582	A > C	ABCB1*2	S893A	0.4765	0.5498	-0.0733	Atazanavir/simvastatin/ondansetron/sunitinib	Decreased clearance
8	18258103	*NAT2*	rs1799930	G > A	NAT2*6	R197Q	0.1394	0.2730	-0.1336	Ethambutol/isoniazid/pyrazinamide/rifampin	Increased risk of hepatoxicity
10	96798749	*CYP2C8*	rs10509681	T > C	CYP2C8*3	K329R	0.0562	0.0838	-0.0276	Rosiglitazone	Increased metabolism
11	17409572	*KCNJ11*	rs5219	T > C	NR	K23E	0.3899	0.6477	-0.2578	Glibenclamide and sulfonamides	Decreased response
	67352689	*GSTP1*	rs1695	A > G	NR	I188V	0.4635	0.3402	0.1233	Cyclophosphamide, epirubicin, uracil-based compounds and platinum-based compounds	Decreased response and increased toxicity
12	48272895	*VDR*	rs2228570	A > G	NR	M51T	0.4988	0.6295	-0.1307	Peginterferon alfa-2b/ribavirin	Decreased response
16	69745145	*NQO1*	rs1800566	G > A	NR	P187S	0.4356	0.2521	0.1835	Anthracyclines and uracil-based compounds	Worse outcome
19	4541207	*APOE*	rs7412	C > T	*APOE-E2*	R176C	0.0447	0.0612	-0.0165	Atorvastatin	Increased response
22	19951271	*COMT*	rs4680	G > A	NR	V158M	0.3924	0.4625	-0.0701	Nicotine/methadone/oxycodone/remifentanil/sufentanil/tramadol	Increased response
**Evidence 2B**											
**Chr**	**bp**	**Gene**	**SNP**	**Alleles**	**Synonymous**	**Amino acid change**	**MAF**	**gMAF**	**ΔMAF**	**Drug**	**Effect on Drug**
1	226019633	*EPHX1*	rs1051740	T > C	NR	Y113H	0.4059	0.3215	0.0844	Carbamazepine	Higher metabolism
	226026406		rs2234922	A > G	NR	H139R	0.0735	0.1870	-0.1135	Carbamazepine	Increased dose
3	124456742	*UMPS*	rs1801019	G > C	NR	G213A	0.3442	0.1946	0.1496	Capecitabine/fluorouracil/leucovorin/tegafur	Increased toxicity
**4**	**2906707**	***ADD1***	**rs4961**	**G > T**	**NR**	**G460W**	**0.1943**	**0.2027**	**-0.0084**	**Furosemide/spironolactone**	**Decreased response**
	69536084	*UGT2B15*	rs1902023	A > C	UGT2B15*2	Y85D	0.3329	0.5149	-0.1820	Lorazepam/oxazepam	Increased clearance
5	7870973	*MTRR*	rs1801394	A > G	NR	I49M	0.1976	0.4678	-0.2702	Methotrexate	Greater toxicicty and increased response
6	154360797	*OPRM1*	rs1799971	A > G	NR	N40D	0.2181	0.1884	0.0297	Naloxone/ethanol/alfentanil/buprenorphine/fentanyl/heroin/morphine/sufentanil/tramadol	Increased cortisol peak and Increased dose (opioids)
	160113872	*SOD2*	rs4880	A > G	NR	V16A	0.3430	0.4823	-0.1393	Cyclophosphamide	Decreased survival
15	78882925	*CHRNA5*	rs16969968	G > A	NR	D398N	0.1192	0.2655	-0.1463	Nicotine	Increased risk of dependence
17	4836381	*GP1BA*	rs6065	C > T	NR	T161M	0.1510	0.0980	0.0530	Aspirin	Decreased risk of resistance
19	44055726	*XRCC1*	rs25487	T > C	NR	Q399R	0.2342	0.6850	-0.4508	Platinum coumpounds	Decreased response
	45912736	*ERCC1*	rs3212986	C > A	NR	Q506K	0.4390	0.2864	0.1526	Platinum-based compounds	Decreased risk of nephrotoxicity
20	3193842	*ITPA*	rs1127354	C > A	NR	P32T	0.0240	0.0750	-0.0510	Peginterferon alfa-2b/ribavirin/interferon alfa-2b	Decreased risk of anemia
21	37518706	*CBR3*	rs1056892	G > A	NR	V244M	0.2512	0.3669	-0.1157	Anthracyclines	Decreased risk of cardiac damage
22	44324727	*PNPLA3*	rs738409	C > G	NR	I148M	0.4174	0.2777	0.1397	Asparaginase/cyclophosphamide/daunorubicin/prednisolone/vincristine	Increased risk of hepatoxicity

^1^MAF, Minor allele frequency in the MxGDAR/Encodat. ^2^gMAF, Global minor allele frequency (MAF) reported by the gnomAD. ^3^ΔMAF, difference between MxDAR/Encodat individuals and gMAF. ^4^ In bold are present the non-significant variants (p-value < 0.05).

### Analysis of Actionable Pharmacogenetic Synonymous, Benign, and Regulatory Variants in the MxGDAR/Encodat

A total of 427 synonymous, benign, and regulatory variants were identified, only seven of which had been previously been associated with drug response (**Table 5**). Of these seven, there was one at evidence level 1A (CYP2C19*2), three at level 2A (*ABCG2* p.Q141K, NAT2*7), and three at level 2B (*GNB3* p.S274S, *GP1BA* p.T161M, *NEDD4L* p.Q8Q). The differences in minor allele frequency between the MxGDAR/Encodat and the gnomAD ranged from -0.1820 to 0.1387. The synonymous variant *GNB3* p.S274S had the largest negative difference (dMAF = -0.1820), and the variant *ABGCG2* p.Q141K the largest positive difference (dMAF = 0.1387).

**Table 5:** Synonymous, benign, and regulatory actionable pharmacogenetic variation in the MxGDAR/Encodat.

Evidence 1A											
Chr	bp	Gene	SNP	Alleles	Synonymous	Amino acid change	MAF^1^	gMAF^2^	ΔMAF^3^	Drug	Effect on Drug
10	96541616	*CYP2C19*	rs4244285	G > A	CYP2C19*2	p.P227P	0.1061	0.2214	-0.1153	Amitriptiline/Esctialopram/Citalopram/Clomipramine/Sertraline/Clopidrogel	Poor Metabolizer (Clinical CPIC Dosing Guideline)
**Evidence 2A**											
4	89052323	*ABCG2*	rs2231142	C > A	NR	p.Q141K	0.2581	0.1194	0.1387	Rosuvastatin	Higer plasma concentration
8	18258370	*NAT2*	rs1799931	G > A	NAT2*7	p.G286E	0.1647	0.0773	0.0874	Ethambutol/isoniazid/pyrazinamide/rifampin	Increased risk of hepatoxicity
16	31105353	*VKORC1*	rs17708472	G > A	VKORC1*4	Promoter (ENSR00000085299)	0.0798	0.0937	-0.0139	Warfarin	Higher dose
**Evidence 2B**											
12	6954875	GNB3	rs5443	C > T	NR	p.S274S	0.6374	0.8194	-0.1820	Sildenafil	Reduction in positive erectile response
17	4836381	GP1BA	rs6065	C > T	NR	p.T161M	0.1511	0.1316	0.0195	Aspirin	Increased aspirin resistance
18	55816791	NEDD4L	rs4149601	G > A	NR	p.Q8Q	0.1340	0.2762	-0.1422	Diuretics or hydrochlorothiazide	Decreased response

^1^MAF, Minor allele frequency in the MxGDAR/Encodat. ^2^gMAF, Global minor allele frequency (MAF) reported by the gnomAD. ^3^ΔMAF, difference between MxDAR/Encodat individuals and gMAF.

### Analysis of Actionable Pharmacogenetic Variants in the Admixture Subgroups

**Figure 3:**
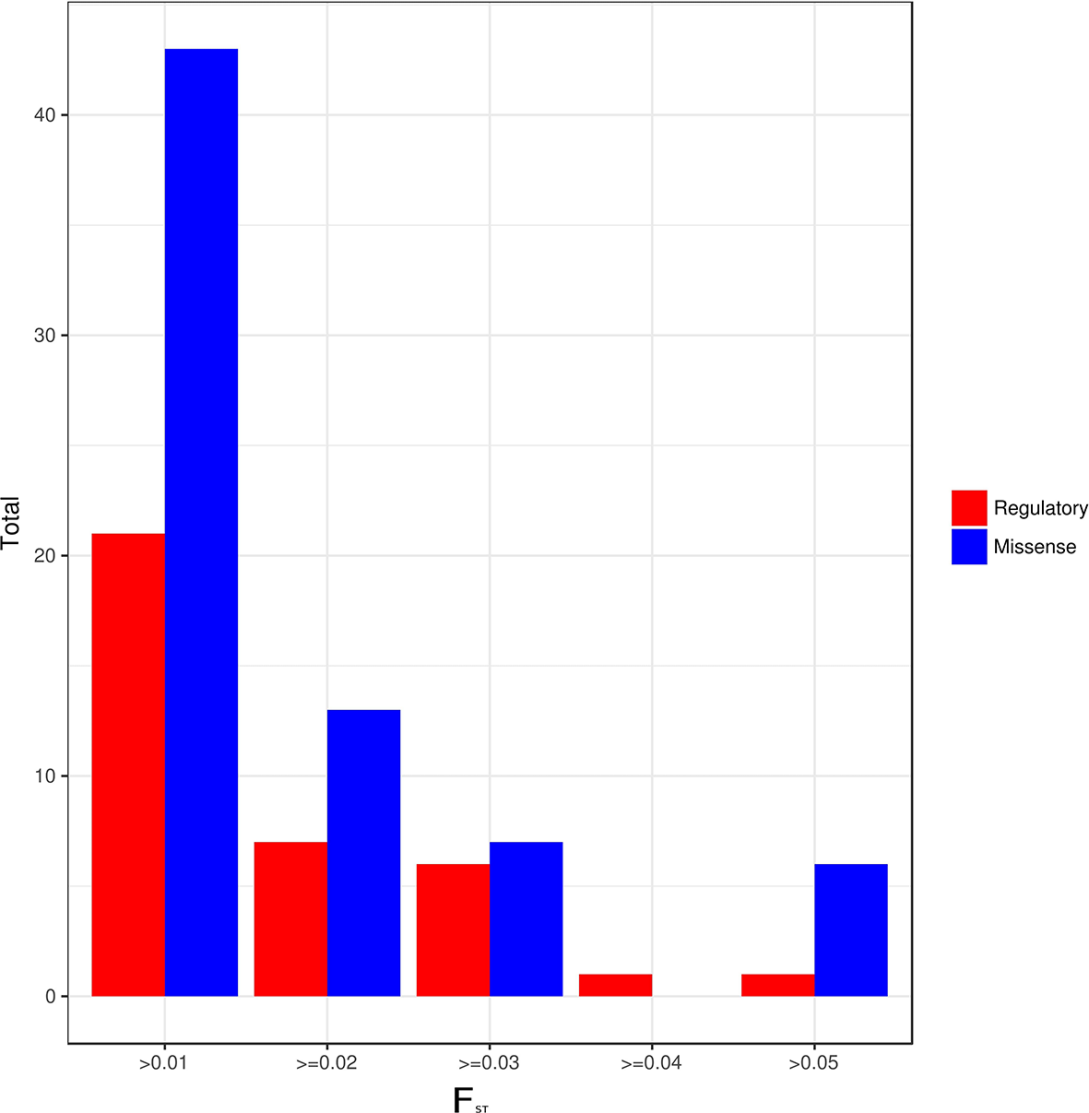
Regulatory and missense variants by F_ST_ value between admixture groups.

**Figure 4:**
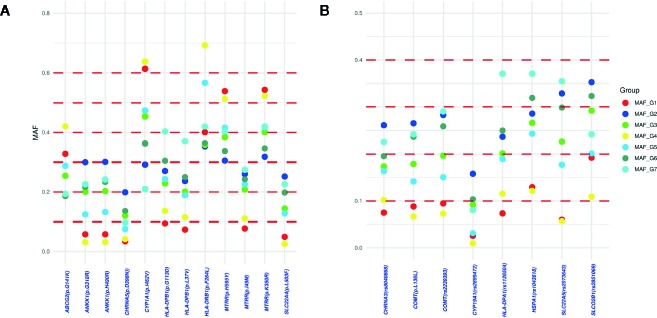
Differences in minor allelic frequencies (MAF) of the PGx variants with F_ST_ > 0.03: **(A)** MAF differences of the damaging missense variants between admixture groups; **(B)** MAF differences of regulatory, benign or synonymous variants between admixture groups.

An analysis of differentiation of the identified actionable pharmacogenetic variants in the different admixture groups found a total of 105 PGx variants with a low degree of differentiation (F_ST_ > 0.01), of which 36 (34.29%) had a regulatory or synonymous function and 69 (65.71%) were missense variants (**Figure 3**). Of the latter, 20 variants had a F_ST_ > 0.03. In Groups 1 and 4, both with regional distribution (one in the Central-East region, and the other in the Southeast; see **Figures 4** and **2C**),15 of the 20 variants had lower frequencies, and five higher frequencies.

## Discussion

The analysis of actionable pharmacogenetic variants in admixed populations has been centered on genes or groups recruited in specific regions (Bonifaz-Peña et al., 2014; Cuautle-Rodríguez et al., 2014; Gonzalez-Covarrubias et al., 2016; Gonzales-Covarrubias et al., 2017). The present study is one of the first to analyze the distribution of known actionable PGx variants in a population-based sample for all states of Mexico, and examining the possible contributions a consideration of admixture patterns could make to pharmacogenetic counseling. The analysis found that 50 actionable PGx variants (96.15%) were different in the MxGDAR/Encodat database than in the gnomAD database for the global population. The following sections will discuss the implications of these differences for the pharmacological parameters of response, toxicity, and dosage requirements, by PharmGKB evidence level.

### Evidence Level 1

The *XPC* p.Q939K variant, with evidence level 1B, reduces toxicity to individuals treated with platinum-based compounds (Caronia et al., 2009; Sakano et al., 2010). It was more than 30% less prevalent in the Mexican population, which could mean increased risk for toxicity. The only variant with evidence level 1 that showed greater MAF in the MxGDAR/Encodat was NUDT15*3 (dMAF = 0.0452), a variant that may increase the risk of leukopenia or neutropenia in those individuals treated with purine compounds (Yang et al., 2014). Differences were also found in CYP2C19*2, which has treatment guidelines that depend on different drug-gene relationships, and which had a lower MAF, which could mean a reduced response to drugs like escitalopram, citalopram, and clopidogrel. The reduction of this allele has been previously reported in a sample from western Mexico (Favela-Mendoza et al., 2015).

### Evidence Level 2

The variants with evidence level 2, *ABCG2* p.Q141K, *MTHFR* p.A22V, *GSTP1* p.I188V, *NQO1* p.P187S, *UMPS* p.G213A, *ERCC1* p.Q506K, and *PNPLA3* p.I148M, showed higher allele frequencies in the Mexican population. These variants could affect the treatment of cancer patients. *MTHFR* p.A22V could have a greater degree of toxicity and decreased response in treatments with methotrexate and platinum-based compounds (Huang et al., 2008; López-Rodríguez et al., 2018), *GSTP1* p.I188V could lessen the response to cyclophosphamide, epirubucin, uracil-based, and platinum-based compounds (Oliveira et al., 2010; Zhang et al., 2011), and *NQO1* p.P187S could diminish the response to anthracyclines and uracil-based compounds (Fagerholm et al., 2008). PNPLA3 p.I148M could increase hepatotoxicity in individuals treated with asparaginase, ciclophosphamide, daunorubicin, prednisolone, or vincristine (Chambers et al., 2011; Gutierrez-Camino et al., 2017), while *ERCC1* p.Q506K could decrease nephrotoxicity in patients treated with platinum-based compounds (Tzvetkov et al., 2011). *UMPS* p.G213A could increase the toxicity of the cancer drugs capacetabine and fluorouracil (Tsunoda et al., 2011) and of leucovirin, an agent used to reduce the effect of methotrexate treatment. *ABCG2* p.Q141K increases the plasma concentration of rosuvastatin, which could reduce its effect in the Mexican population (Tomlinson et al., 2010).

The variants NAT2*6, *KCNJ11* p.K23E, *VDR* p.M51T, *EPHX1* p.H139R, UGT2B15*2, *MTRR* p.I49M, *SOD2* p.V16A, *CHRNA5* p.D398N, *XRCC1* p.Q399R, GNB3 p.S274S, *NEDD4L* p.Q8Q, and *CBR3* p.V244M all had reduced MAF in the Mexican population. These variants could potentially affect a variety of drugs, including those for cancer, psychiatric illness, tuberculosis, viruses, and diabetes. The reduction in the frequency of the NAT2*6 allele could reduce hepatotoxicity in treatment with ethambutol, pyramizid, and rifampin (Kim et al., 2009; Ben Mahmoud et al., 2012), and *KCNJ11* p.K23E could increase the response to glibenclamide and sulfonamides (Javorsky et al., 2012). The reduced MAF of *VDR* p.M51T could increase response rates to peginterferon and ribavirin (García-Martín et al., 2013; El-Derany et al., 2016). The reduction of *EPHX1* p.H139R could result in lower dose requirements for the psychiatric drug carbamazepine (Kamiya et al., 2005; Hung et al., 2012; Puranik et al., 2013), and that of UGT2B15*2 could reduce the clearance of lorazepam and oxazepam (He et al., 2009; Bhatt et al., 2019). For oncology patients, *MTRR* p.I49M could reduce the toxicity of metrotrexate (López-Rodríguez et al., 2018), *XRCC1* p.Q399R could increase the response to platinum-based compounds (Yin et al., 2012), and *SOD2* p.V16A could increase survival in those treated with cyclophosphamide (Glynn et al., 2009). *CBR3* p.V244M could increase the risk of cardiac damage from anthracyclines (Blanco et al., 2008; Gonzalez-Covarrubias et al., 2008). Interestingly, the variant *CHRNA5* p.D398N could reduce the dependence on nicotine in the Mexican population (Johnson et al., 2010; Gelernter et al., 2015; Hancock et al., 2017), which could help to explain the lower rates of cigarette consumption reported for Latino populations (Pagano et al., 2018). Further studies are needed to explore the effect of this variant on nicotine dependence.

### The Pharmacogenetic Counseling Based on these Variants Could Depend on the Admixture Pattern Found in different Geographical Regions of Mexico

The admixture of the Mexican population has been divided into two populations: Native Americans (NA) and Mexican Mestizos (MM) (Wang et al., 2008; Moreno-Estrada et al., 2014). There have been some attempts to identify how variants in the NA population affect pharmacology (Gonzalez-Covarrubias et al., 2016; Romero-Hidalgo et al., 2017; Gonzalez-Covarrubias et al., 2019), but in many analyses the entire MM population is grouped together, with no estimation of how admixture patterns within that group could affect the frequency of actionable pharmacogenetic variants or clinical counseling.

In the present study, a global ancestry model-based clustering algorithm identified seven admixture groups, some of which had a particular distribution in specific geographic areas in Mexico. Groups 4 and 5 were found only in the Central-East and Southeast, and Group 1 in the Northwest, North, and Central-West regions. Groups 1 and 4 are the most closely related to the NA population. The mapping of geographically dependent admixture patterns has been analyzed in the African-American and Latino populations in the United States (Bryc et al., 2015). Bryc et al., found that specific admixture groups of these populations were more prevalent in some regions, and they also reported that self-identification (used mainly in epidemiological studies) did not correlate with individuals’ ancestry. The identification of admixture patterns could help to more accurately identify individual ancestry and aid with pharmacogenetic counseling and treatment decisions guided by genomics.

The present study found 20 actionable Pgx variants with possible dependence on admixture patterns. The variants *MTRR* p.I49M and NAT2*6 are less frequent in individuals from Groups 1 and 4 (those with a greater degree of NA ancestry) and could predict a reduction in toxicity in cancer patients treated with methotrexate, reduce the risk of hepatotoxicity in tuberculosis patients, and the risk in its use for treatment of acute lymphoblastic leukemia (Gast et al., 2007; Huang et al., 2008), for which it is one of the main drugs used in Mexico (Medellin-Garibay et al., 2019). Another variant with a substantial difference in MAF between admixture groups was *ABCG2* p.Q141K, which affects the response to rosuvastatin (Tomlinson et al., 2010), the most cost-effective option for dyslipidemia treatment in Mexico (Briseño and Mino-León, 2010).

Although we were able to identify missense variants in pharmacogenes with possible differential actionable effects in the Mexican population, one limitation of this study is that the MxGDAR/Encodat database does not have information about drug responses or adverse reactions, which limits the conclusions that can be drawn about the specific effects on the Mexican population. Another limitation is our use of a genotyping platform with reduced coverage and a fixed number of variants. Microarray genotyping technology eliminates the possibility of finding new variants in these pharmacogenes, but this could be accomplished with other techniques, such as next-generation technologies (NGS). An analysis of the Mexican population recently performed utilizing NGS found novel variation for some pharmacogenes between NA and MM populations (Gonzalez-Covarrubias et al., 2019). However, that analysis considered the MM population as a single group: an analysis of admixture patterns within the MM population, as performed in the present study, could be applied using NGS to refine the analysis of the Mexican population, and genomic guided some public health decisions in Mexico.

## Conclusions

A description of the pharmacogenetic variants that can be actionable in this representative subsample of the Mexican population will help to understand and reduce treatment disparities in persons of admixed genetic background with differing pharmacogenetic variants. Such studies can serve as guides to precision medicine in the Mexican population and in other populations with mixed genetic backgrounds.

## Data Availability Statement

The datasets generated for this study can be found in the European Variation Archive (EVA). 

## Ethics Statement

The studies involving human participants were reviewed and approved by Instituto Nacional de Psiquiatría Ramón de la Fuente Muñiz and Instituto Nacional de Medicina Genómica. Written informed consent to participate in this study was provided by the participants’ legal guardian/next of kin.

## Author Contributions

JM-M, AG-M, JV, and HN developed the analyses and wrote the first version of the manuscript. JM-M, RM and JV performed bioinformatics and statistical analyses. JV, RM, CF, MB, ER, and MM-M contributed to data collection. BC, AA, JM-M, and AG-M contributed to the genetic experiments. HN, MM-M and JV conceived, designed, and coordinated the project. 

## Funding

This study received funding from the Instituto Nacional de Medicina Genómica (Grant No. 23/2015/I), and from the Comisión Nacional de Ciencia y Tecnología (CONACyT) 2016 Fund for the Development of Scientific Projects to Address National Problems (Grant No. PN22296). The development of the surveys was funded by the Comisión Nacional Contra las Adicciones (CONADIC).

## Conflict of Interest

The authors declare that the research was conducted in the absence of any commercial or financial relationships that could be construed as a potential conflict of interest.

## Supplementary Material

Supplementary File 1 Variants filtered-out during quality controls steps.
